# Ventilator associated pneumonia in COVID-19 patients: A retrospective cohort study

**DOI:** 10.5578/tt.20239906

**Published:** 2023-03-10

**Authors:** L. FERLİÇOLAK, E. SARICAOĞLU, B. BİLBAY, N. D. ALTINTAŞ, F. YÖRÜK

**Affiliations:** 1 Division of Intensive Care, Department of Internal Medicine, Ankara University Faculty of Medicine, Ankara, Türkiye; 2 Department of Infection Diseases and Clinical Microbiology, Ankara University Faculty of Medicine, Ankara, Türkiye; 3 Deparment of Internal Medicine, Ankara University Faculty of Medicine, Ankara, Türkiye

**Keywords:** COVID-19, intensive care unit, ventilator associated pneumonia, Acinetobacter spp, mortality

## Abstract

**ABSTRACT:**

Ventilator associated pneumonia in COVID-19 patients: A retrospective cohort study

**Introduction:**

We aimed to evaluate ventilator-associated pneumonia (VAP) incidence rate, risk factors, and isolated microorganisms in COVID-19 patients as the primary endpoint. Evaluation of VAP-associated intensive care unit (ICU) and hospital mortalities was the secondary endpoint.

**Materials and Methods:**

Records of patients admitted between March 2020- June 2021 to our pandemic ICU were reviewed and COVID-19 patients with VAP and non-VAP were evaluated retrospectively. Comorbidities, management, length of ICU stay, and outcomes of VAP and non-VAP patients, as well as risk factors for VAP mortality, were identified.

**Results:**

During the study period, 254 patients were admitted to the ICU. After the exclusion, the data of 208 patients were reviewed. In total, 121 patients required invasive mechanical ventilation, with 78 (64.5%) developing VAP. Length of ICU and hospital stays were longer in VAP patients (p< 0.01 and p< 0.01 respectively). Steroid use was higher in VAP patients, although it was not statistically significant (p= 0.06). APACHE II score (p< 0.01) was higher in non-VAP patients. ICU mortality was high in both groups (VAP 70%, non-VAP 77%). VAP mortality was higher in males (p= 0.03) and in patients who required renal replacement therapy (p= 0.01). Length of ICU stay (p= 0.04), and length of hospital stay (p< 0.01) were both high in VAP survivors. The most common isolated microorganisms were Acinetobacter spp. and Klebsiella spp. in VAP patients and most of them were extensively drug-resistant.

**Conclusion:**

Critically ill COVID-19 patients who required invasive mechanical ventilation developed VAP frequently. The length of ICU stay was longer in patients who developed VAP and ICU mortality was high in both VAP and non-VAP patients. The length of hospital and ICU stays among VAP survivors were also considerably high which is probably related to the long recovery period of COVID-19. The most frequently isolated microorganisms were Acinetobacter spp. and Klebsiella spp. in VAP patients

## Abstract (tr)

**ÖZ:**

COVID-19 hastalarında ventilatör ilişkili pnömoni: Retrospektif kohort çalışması

**Giriş:**

Çalışmamızda primer sonlanım noktası olarak COVID-19 hastalarında ventilatör ilişkili pnömoni (VİP) insidansını, risk faktörlerini ve izole edilen mikroorganizmaları değerlendirmeyi amaçladık. VİP ile ilişkili yoğun bakım ünitesi (YBÜ) ve hastane ölümlerinin değerlendirilmesi ikincil sonlanım noktasıydı.

**Materyal ve Metod:**

Mart 2020 ve Haziran 2021 tarihleri arasında pandemi yoğun bakım ünitemize başvuran hastaların kayıtları incelenerek VİP olan ve olmayan COVID-19 hastaları retrospektif olarak değerlendirildi. VİP mortalitesi için risk faktörlerinin yanı sıra VİP olan ve olmayan hastaların komorbiditeleri, kullanılan tedaviler, YBÜ kalış süresi ve sonlanımları kaydedildi.

**Bulgular:**

Çalışma süresi boyunca 254 hasta yoğun bakıma kabul edildi. Dışlamadan sonra 208 hastanın verileri incelendi. Toplam 121 hastada invaziv mekanik ventilasyon uygulanmıştı ve 78 (%64.5) hastada VİP gelişmişti. VİP hastalarında yoğun bakım ve hastanede kalış süreleri daha uzundu (sırasıyla p< 0.01 ve p< 0.01). İstatistiksel olarak anlamlı olmamakla birlikte VİP hastalarında steroid kullanımı daha yüksekti (p= 0.06). APACHE II skoru (p< 0.01) VİP gelişmeyen hastalarda daha yüksek saptandı. YBÜ mortalitesi her iki grupta da yüksekti (VİP %70, VİP dışı %77). VİP mortalitesi erkeklerde (p= 0.03) ve renal replasman tedavisi gereken hastalarda (p= 0.01) daha yüksekti. Yoğun bakımda kalış süresi (p= 0.04) ve hastanede kalış süresi (p< 0.01) sağ kalanlarda yüksekti. En sık izole edilen mikroorganizmalar Acinetobacter spp. ve Klebsiella spp. olarak izlendi ve bunların çoğu bir veya iki antibiyotik grubu hariç, hepsine dirençli mikroorganizmalardı

**Sonuç:**

İnvaziv mekanik ventilasyona ihtiyaç duyan kritik COVID-19 hastalarında sıklıkla VİP geliştiği izlendi. VİP gelişen hastalarda yoğun bakımda kalış süresi uzundu ve hem VİP olan hem de olmayan hastalarda YBÜ mortalitesi yüksekti. Sağ kalan VİP hastalarında hastanede ve yoğun bakımda kalış süreleri de oldukça yüksekti, bu muhtemelen COVID-19’un uzun iyileşme süresiyle ilişkilidir. En sık izole edilen mikroorganizmalar Acinetobacter türleri ve Klebsiella türleri olmuştur.

## Introduction

Ventilator-associated pneumonia (VAP) is defined as an infection of the pulmonary parenchyma that develops in people who require mechanical ventilation for at least 48 hours ( 1 ). Despite advances in antimicrobial therapy, enhanced supportive care, and preventive measures, VAP remains an important cause of morbidity and mortality, impeding the course of approximately 10% of patients with invasive mechanical ventilation requirement, with a predicted mortality rate of 13% ( 2 ).

The Coronavirus disease-2019 (COVID-19), which prompts systemic inflammation and mostly leads to pneumonia, is a global pandemic. Overall, almost 25% of COVID-19 patients were admitted to the intensive care unit (ICU) with respiratory failure requiring invasive mechanical ventilation ( 3 , 5 ). While the ICU mortality rate was reported to be up to 60%, the mortality rate might be even higher in mechanically ventilated patients (24-80%) ( 3 , 4 , 6 ). Significantly higher VAP-associated lower respiratory tract infection incidence in COVID-19 patients was reported in a multicenter cohort study ( 7 ). VAP was frequently reported in patients with COVID-19-associated acute respiratory distress syndrome (ARDS) ( 8 ). Mortality rates were also higher in COVID-19 patients with VAP ( 9 ).

Although there have been numerous studies on VAP in COVID-19 patients, we believe that patients’ outcomes may vary depending on the centers’ local resistance patterns of microorganisms and the experience of the team of specialists. Because there are few effective treatments for infections caused by multidrug-resistant (MDR) microorganisms, increasing understanding of local epidemiology, risk stratification of patients, and infection control measures remain critical in the management of VAP ( 10 ).

The purpose of this study was to define VAP frequency in a COVID-19 ICU, as well as to evaluate comorbidities, management, and outcomes of VAP and non-VAP patients, as well as risk factors for VAP mortality in COVID-19. We also identified the most common microorganisms isolated from VAP patients.

## MATERIALS and METHODS

This observational retrospective study was conducted in a university hospital pandemic intensive care unit between March 17^th^, 2020, and June 1^st^, 2021. Adult patients who were admitted to the ICU with positive nasopharyngeal swabs and/or deep tracheal aspirates for SARS-CoV-2 polymerase chain reaction were included in the study. This study was approved by the local ethics committee on August 25, 2022 (no: 2022/452).

### Definitions

VAP is suspected when patients develop two of the following signs and symptoms after at least 48 h of mechanical ventilation: (i) new onset of fever (≥38≥) or hypothermia (≤35≥); (ii) new onset or changing characteristics of respiratory secretions; (iii) leukocytosis or leucopenia; (iv) increased minute ventilation; (v) arterial oxygenation decline; (vi) increased vasopressor infusion requirement to maintain target blood pressure; (vii) new or progressive persistent infiltrate on chest X-ray or computed tomography. Patients’ data were collected only for the first VAP episode.

Deep endotracheal aspirate cultures and microscopic evaluations were assessed if the results were significant for VAP.

Multidrug-resistant (MDR) isolates were described as acquired non-susceptibility to at least one agent in three or more antimicrobial categories. Extensively drug-resistant (XDR) isolates were described as nonsusceptibility to at least one agent in all but two or fewer antimicrobial categories. Pan-drug resistant (PDR) isolates were described as non-susceptibility to all antimicrobial categories.

#### Data Collection

Patients’ age, gender, comorbidities, acute physiological and chronic health evaluation (APACHE) II score at admission, sequential organ failure assessment (SOFA) score, partial arterial oxygen-fractional inspired oxygen (PaO_2_/FiO_2_) ratio, and immunocompromised status were documented. Drugs used for COVID-19 treatment (steroids, anakinra, and tocilizumab) were also recorded. Microorganisms isolated from deep endotracheal aspirate cultures and their resistance patterns were reported.

Length of ICU and hospital stays, ICU mortality, and hospital mortalities were defined as outcome parameters.

#### Statistical Analysis

SPSS (IBM, Armonk, NY, USA) version 25.0 was used for analyses. Quantitative variables were expressed as medians (interquartile range) and categorical variables were expressed as numbers (percentage). Patient characteristics were described according to study groups (with and without VAP) first and then VAP patients (survivor and non-survivor) with formal statistical comparisons. Multiple logistic regression analysis was performed to explore the characteristics associated with mortality in COVID-19 patients who developed VAP in the ICU. Parameters included in the multivariate analysis were selected according to univariate analysis results (p< 0.1). A p value of < 0.05 was considered statistically significant.

### RESULTS

The demographic and clinical characteristics of VAP and non-VAP COVID-19 patients are shown in Table 1. The median age was 70 years (25-75 percentiles 62-77) and 61% were male (74/121). The most frequent comorbid conditions were hypertension (73/121; 60%) and diabetes mellitus (51/121; 42%). All our study patients had severe ARDS when admitted to the ICU (median PaO2/FiO2 ratio was 92). In the VAP group, the median age was 69 (63-77) and 61% were males (48/78). There was no difference between VAP and non-VAP groups according to age, gender, and comorbidities. Median Charlson comorbidity index and SOFA score were similar, on the other hand, the median APACHE II score was higher in patients without VAP (p= 0.02). The length of ICU and hospital stay was longer in VAP patients (p< 0.01 and p< 0.01, respectively). ICU and hospital mortality rates were similar in VAP and non-VAP patients.

Table 2 shows a descriptive analysis of the ICU outcomes of COVID-19 patients with VAP (survivor vs. non-survivor). In terms of age, comorbidities, severity scores, and treatment modalities, survivors and non-survivors were comparable. Male gender and renal replacement therapy requirement were higher in the non-survivor VAP group (p= 0.03 and p= 0.01, respectively). In addition, the length of ICU and hospital stays were longer in VAP survivors (p= 0.04 and p< 0.01, respectively).

Except for one patient, whose culture was obtained using bronchoalveolar lavage, all cultures were obtained with deep tracheal aspiration. Table 3 shows the isolated pathogens and their resistance patterns. Endotracheal aspirate cultures from one patient revealed three different microorganisms, while endotracheal aspirate cultures from 22 patients revealed two different microorganisms simultaneously. Acinetobacter spp. (41, 5%) and Klebsiella spp. (38, 49%) were the most frequently identified microorganisms and most of them were XDR strains.

**Table 1:** Demographic variables, comorbidities, management and outcomes of VAP and non-VAP patients

	Total (n= 121)	VAP (n= 78)	Non-VAP (n= 43)	p
Age (years)^*^	70 (62-77)	69 (63-77)	71 (60-78)	0.94
Male gender^**^	74 (61)	48 (61)	26 (60)	0.90
APACHE II^*^	21 (16-27)	19 (15-25)	25 (16-33)	0.02
SOFA^*^	5 (3-7)	4 (3-7)	5 (3-10)	0.99
CCI ^*^	5 (3-6)	5 (3-6)	5 (3-7)	0.15
Comorbidities ^**^			
HT	73 (60)	46 (59)	27 (63)	0.68
DM	51 (42)	36 (46)	15 (35)	0.23
CAD	48 (40)	31 (40)	17 (40)	0.98
Malignancy	28 (23)	16 (20)	12 (27)	0.35
COPD	20 (16)	14 (18)	6 (14)	0.57
CHF	22 (18)	12 (15)	10 (23)	0.28
Immunsupression	13 (11)	7 (9)	6 (13)	0.39
CVD	13 (11)	7 (9)	6 (13)	0.39
CRF	10 (8)	8 (10)	2 (4)	0.28
PaO_2_/FiO_2_^*^	92 (60-132)	86 (58-128)	100 (60-140)	0.52
Steroids ^**^	83 (68)	58 (74)	25 (58)	0.06
Anakinra^**^	16 (13)	13 (16)	3 (7)	0.13
Tocilizumab ^**^	4 (3)	3 (4)	1 (8)	0.65
RRT ^**^	27 (22)	18 (23)	9 (20)	0.78
Length of hospital stay before ICU*	4 (1-9)	4 (1-8)	5 (1-12)	0.26
Length of ICU stay*	20 (8-28)	25 (17-33)	7 (3-14)	<0.001
Total length of hospital stay ^*^	22 (10-36)	27 (19-43)	7 (3-16)	<0.001
ICU mortality ^**^	88 (72)	55 (70)	33 (77)	0.46
Hospital mortality ^**^	97 (80)	63 (81)	34 (79)	0.82

VAP: Ventilator associated pneumonia, APACHE II: Acute physiological and chronic health evaluation score, SOFA: Sequential organ failure score, CCI: Charlson comorbidity index, HT: Hypertension, DM: Diabetes mellitus, CAD: Coronary artery disease, COPD: Chronic obstructive pulmonary disease, CHF: Chronic heart failure, CVD: Cerebrovascular disease, CRF: Chronic renal failure, RRT: Renal replacement therapy. ^*^median (25-75 quartiles), ^**^n (%).

### DISCUSSION

COVID-19 patients are more susceptible to infections for several reasons. COVID-19, as well as the medications used to treat it, induces an immunosuppressive state. Furthermore, the prevalence of ARDS is increased in COVID-19 patients, increasing the likelihood of VAP development. VAP is more common in severe COVID-19 patients who require invasive mechanical ventilation. In a large cohort of 4244 critically ill patients with COVID-19 from the UK, Schmidt et al. reported 58% VAP development ( 11 ). In a French multicenter study, the VAP incidence rate was reported as 48.9% ( 12 ). In another study that compared COVID-19-ARDS and non-COVID-19- ARDS patients, they also found a higher incidence of

VAP in COVID-19-ARDS patients with a rate of 62% ( 8 ). In a European multicenter cohort study, ventilatorassociated lower respiratory tract infections were reported frequently in COVID-19 patients %50.5) ( 7 ). Our study also showed a high incidence rate of VAP (64.5%) in COVID-19 patients.

In the coVAPid cohort, VAP was associated with longer ICU stay ( 13 ). We also observed that patients with VAP had a longer ICU stay (median 25 days).

Immunosuppressive treatments such as dexamethasone, and interleukin one and six antagonists, which were used frequently in the treatment of COVID-19 patients who required oxygen support, might pose a risk of VAP development. In our study, 83 (68%) patients were treated with corticosteroids and 58 (74%) of them developed VAP.

**Table 2:** Demographic variables, comorbidities, management and outcomes of VAP survivors and non-survivors

	VAP (n= 78)	Survivor (n= 23)	Non-survivor (n= 52)	p
Age (years) ^*^	69 (63-77)	67 (54-77)	70 (64-77)	>0.999
Male gender ^**^	48 (61)	10 (43)	38 (73)	0.03
APACHE II ^*^	19 (15-25)	18 (15-23)	19.5 (15-26)	0.72
SOFA ^*^	4 (3-7)	4 (2-6)	5 (3-7)	0.17
CCI ^*^	5 (3-6)	4 (2-5)	5 (3-6)	0.48
Comorbidities ^**^				
HT	46 (59)	12 (52)	34 (65)	0.43
DM	36 (46)	9 (27)	27 (52)	0.42
CAD	31 (40)	8 (35)	23 (44)	0.56
Malignancy	16 (20)	5 (21)	11 (21)	0.86
COPD	14 (18)	2 (9)	12 (23)	0.16
CHF	12 (15)	5 (21)	7 (13)	0.31
Immunsupression	10 (12)	3 (13)	7 (13)	0.97
CVD	7 (9)	2 (9)	5 (10)	0.95
CRF	8 (10)	1 (4)	7 (13)	0.26
PaO2/FiO2 ^*^	86 (58-128)	91 (67-132)	83 (55-136)	0.56
Steroids ^**^	58 (74)	16 (70)	42 (77)	0.53
Anakinra ^**^	13 (16)	6 (26)	7 (11)	0.14
Tocilizumab ^**^	3 (4)	1 (4)	2 (4)	0.88
RRT^**^	18 (23)	1 (4)	17 (30)	0.01
Length of hospital stay before ICU^*^	4 (1-8)	1 (0-6)	4 (1-9)	0.32
Length of ICU stay^*^	25 (17-33)	27 (20-38)	23 (16-33)	0.04
Total length of hospital stay^*^	27 (19-43)	46 (33-69)	23 (15-33)	<0.001

VAP: Ventilator associated pneumonia, APACHE II: Acute physiological and chronic health evaluation score, SOFA: Sequential organ failure score, CCI: Charlson comorbidity index, HT: Hypertension, DM: Diabetes mellitus, CAD: Coronary artery disease, COPD: Chronic obstructive pulmonary disease, CHF: Chronic heart failure, CVD: Cerebrovascular disease, CRF: Chronic renal failure, RRT: Renal replacement therapy. ^*^median (25-75 quartiles), ^**^n (%).

**Table 3:** Endotracheal aspirate culture growths and their resistance patterns

	MDR	XDR	PDR	Total
Acinetobacter spp. (n)	2	34	5	41
Klebsiella spp. (n)	5	25	6	38
Pseudomonas spp. (n)	3	1	1	9
Corynebacterium spp. (n)				9
Staphylococcus aureus (n)	2			3

MDR: Multidrug resistant, XDR: Extensively drug resistant, PDR: Pandrug resistant.

VAP rate was higher in patients who received corticosteroids, but it did not reach a statistical significance (p= 0.06). This result could be due to the small number of patients. In a multicenter study from France, it was revealed that dexamethasone treatment was not a risk factor for VAP development ( 14 ). On the other hand, in a propensity-matched retrospective cohort study from Italy, they reported an increased risk of VAP with dexamethasone treatment ( 15 ). We also used IL-1 antagonist (in 16 patients, 13%) and IL-six antagonist (in four patients, 3%) in severe COVID-19 patients. There was no significant increase in VAP rate in those patients, although the number of patients was very small to draw any definitive conclusions. In this regard, studies aimed to assess the efficacy of IL-6 antagonists in COVID-19 patients found no increase in secondary bacterial infections or VAP ( 16 , 17 ).

A multicenter study from Italy reported a mortality rate of 46% in COVID-19 patients with VAP. They also reported the presence of ARDS at the VAP onset as a mortality risk ( 17 ). In our study, the ICU mortality rate was 70% in VAP patients and all of our patients were admitted to ICU with severe ARDS. However, in non-VAP patients, APACHE II scores were higher than in VAP patients. This could be the result of the more severe COVID-19 illness in the non-VAP group. Hence, we did not observe any mortality difference between VAP and non-VAP patients. Likewise, Rouyer et al. reported no mortality difference between patients with or without VAP ( 18 ).

Male gender and renal replacement therapy requirements were higher in the non-survivor VAP group, which are also well-known risk factors for mortality in COVID-19 patients. In addition, the length of ICU and hospital stays were longer in VAP survivors probably due to the prolonged recovery period of COVID-19. Because of its multisystemic and unique nature, the signs of COVID-19 could be traced when studying this patient population.

Acinetobacter spp. and Klebsiella spp. were the main pathogens identified in our study. Most European countries reported Pseudomonas spp. as the most common microorganism in COVID-19 patients ( 7 , 12 , 19 , 20 ). In another study from Türkiye, Rollas et al. reported Acinetobacter baumanii as the leading cause of VAP ( 21 ). Gram-negatives appeared to be the most predominant agents in COVID-19 patients with VAP.

The majority of the isolated microorganisms in this study were XDR (60%). The overall incidence of MDR infections increased by 23.9 percent in a Brazil cohort during the COVID-19 pandemic, and the pandemic was associated with an increase in carbapenemresistant Acinetobacter baumanii infections in both ICU and non-ICU patients ( 22 ). However, a multicenter European study found that MDR microorganisms were less prevalent in COVID-19 patients ( 7 ). Highly resistant bacteria in our study might partially explain the high mortality rates. Our study was conducted during the first months of the pandemic when ICUs were overpopulated, had insufficient personal protective equipment and healthcare personnel availability, and infection control procedures and antimicrobial policies were difficult to implement. These extraordinary circumstances undoubtedly contributed to increased infection rates, resistance to pathogens, and mortalities.

Our study had some limitations. For instance, it was a single-center study, therefore the findings could not be extrapolated. Second, because it was a retrospective analysis and we were unable to reach all antimicrobial treatments, there was no remark on antibiotic medication. Thirdly, the study site was a university hospital where more severe patients with various comorbidities were admitted, which could have an unfavorable effect on the outcomes. Finally, the study’s limited sample size made it difficult to draw conclusions.

### CONCLUSION

VAP was frequently observed in COVID-19 patients who required invasive mechanical ventilation. Length of ICU and hospital stays were longer in VAP patients. Highly resistant Acinetobacter spp. and Klebsiella spp. were the main pathogens isolated in VAP. Mortality rates were high in both VAP and non-VAP COVID-19 patients

#### Ethical Committee Approval:

Ethics committee approval was obtained from Ankara University Human Research Ethics Committee (Decision no: 107-467-22, Date: 02.09.2022).

### CONFLICT of INTEREST

The authors declare that they have no conflict of interest.

### AUTHORSHIP CONTRIBUTIONS

Concept/Design: LF, BB, EMS

Analysis/Interpretation: LF, BB

Data acqusition: LF, BB

Writing: LF, EMS, NDA, FY

Clinical Revision: LF, EMS, FY

Final Approval: NAY, NT, AK, BBK
